# Clinical course and spectrum of intensive care unit patients reactivating herpes simplex-1 virus: A retrospective analysis

**DOI:** 10.4103/0972-5229.45073

**Published:** 2008

**Authors:** Krishna M. Sundar, Karl A. Ludwig, William T. Alward, Michael J. Pearce, Clark T. Bishop, Roy C. Hammond, David R. Hillyard, Steven W. Freestone, Anne Ozment, Barbara C. Cahill

**Affiliations:** **From:**1 Department of Medicine, Utah Valley Reginal Medical Center, Provo; Department of Medicine, University of Utah Medical Center, Salt Lake City, Utah; 2 Department of Respiratory Care, Utah Valley Regional Medical Center, Provo; 3 Department of Medicine, Utah Valley Reginal Medical Center, Provo; 4 Department of Medicine, Utah Valley Reginal Medical Center, Provo; 5 Department of Medicine, Utah Valley Reginal Medical Center, Provo; 6 Department of Radiology, Utah Valley Reginal Medical Center, Provo; 7 Department of Medicine, University of Utah Medical Center, Salt Lake City, Utah; 8 Department of Pathology, Utah Valley Reginal Medical Center, Provo; 9 Department of Pathology, Utah Valley Reginal Medical Center, Provo; 10 Department of Medicine, University of Utah Medical Center, Salt Lake City, Utah

**Keywords:** Herpes simplex-1 virus, pneumonia, viral, ventilator associated

## Abstract

**Background:**

Herpes simplex-1 virus (HSV-1) reactivation in the respiratory tract is common in intensive care unit (ICU) patients. However, susceptible ICU populations are poorly defined. Clinical recognition of HSV infection of the respiratory tract is difficult and the impact of such reactivation is not understood.

**Materials and Methods:**

A retrospective analysis of HSV-1 positive patients encountered over a 5-year period at a multispecialty ICU was carried out. HSV-1 was identified in respiratory secretions using a qualitative polymerase chain reaction (PCR) technique. Patient charts were reviewed for clinical features that would typify HSV-1 respiratory involvement, and the morbidity and mortality risks found with HSV-1 respiratory involvement.

**Results:**

A review of 48 HSV-1 positive ICU patients showed that patients reactivating HSV in the respiratory tract fell into one of the three categories: (1) septic elderly patients with and without ARDS, (2) immunosuppressed patients, especially those receiving high-dose steroids, and (3) post-thoracotomy patients. Abnormalities suggestive of HSV-1 reactivation in the respiratory tract included, haemorrhagic or excessive respiratory secretions, concomitant orofacial herpes (42%), and bronchoscopic abnormalities (hemorrhagic ulcers and mucosal friability) (83%). Twenty eight percent of the HSV-1 infected patients experienced postextubation stridor. HSV-1 reactivation was associated with extended ventilator stays, significant mortality (42%), and ventilator-associated pneumonias (52%).

**Conclusions:**

Identification of susceptible populations and definition of clinical features of HSV-1 related respiratory disease can enable diagnosis of HSV-1 infection in ICU patients. Although detection by a PCR technique can rapidly diagnose HSV-1 reactivation, prospective studies are required to clarify HSV disease versus mere shedding, and understand the impact of HSV-1 reactivation in hospitalized patients.

## Introduction

The viruses from the herpes family are characterized by their ability to remain latent within tissues following initial infection and a tendency to reactivate at mucocutaneous sites.[1] Reactivation of herpes simplex-1 virus (HSV-1) in the orofacial region is a common problem; other sites of herpetic infection include the respiratory tract and gastrointestinal tract.[1] HSV-1 reactivation pneumonitis and esophagitis are well recognized in immunosuppressed patients.[2] However, a number of critically ill and hospitalized patients also develop herpetic infection in the respiratory tract that is infrequently recognized. This study was carried out to identify circumstances in which herpetic reactivation occurs in ICU patients, recognize patterns of respiratory disease, and evaluate the clinical impact of such reactivation.

## Materials and Methods

Hospital records from ICU patients who tested positive for HSV-1 in their respiratory secretions between 2003 and 2007 were reviewed. HSV in sputum, endotracheal aspirate, bronchoscopic aspirates, and lavage fluid (BAL) was identified with a polymerase chain reaction (PCR) method using the Nanogen MGB Alert HSV 1, 2 qualitative assay (Nanogen Inc., Wothell, WA, USA). This PCR method has been validated for HSV-1 and HSV-2 detection in a variety of sample types including respiratory secretions.[3] This assay targets a 179-bp region from the *glycoprotein D* gene and utilizes UTP/UNG contamination control and hybridization probe chemistry allowing for post PCR melting analysis and accommodation of sequence polymorphisms. It also employs a noncompetitive internal control in every sample PCR to monitor for extraction efficiency and potential PCR inhibition.[3] Clinical samples are extracted using the QIAGEN 96-well blood kit, with minor modifications to optimize extraction efficiency. The test was performed in a closed-tube format using a standard-master mix kit (Roche Molecular Biochemicals, Indianapolis, Indiana, USA) and has an analytic sensitivity of approximately 400 HSV genomes per milliliter of clinical fluid [3]. Since this assay does not distinguish between HSV1 and HSV2, further references to HSV detection in respiratory secretions will be meant to imply HSV1 detection. HSV2 infections in the respiratory tract have been reported very rarely in the existing literature.[4]

In patients screened for HSV-1 in the respiratory tract, no *apriori* testing algorithms were used. In fact, no specific recommendations exist for HSV-1 screening except in instances of suspicion of herpetic tracheobronchitis.[5] Previous reports indicate that herpetic tracheobronchitis can be suspected during bronchoscopy when the findings of hemorrhagic mucosal ulcers are encountered.[5] Many patients in this study however lacked the classic bronchoscopic features and manifested a spectrum of clinicoradiologic abnormalities.

Among patients with HSV-1 in respiratory secretions, data was reviewed for demographic information, underlying medical diagnoses, time to HSV diagnosis, bronchoscopic findings, concomitant infections, hospital course, and outcome. Attempt was made to identify diseases or interventions that may have predisposed patients to herpetic reactivation. Charts were reviewed for specific mention of orofacial lesions, appearance of respiratory secretions, and chest auscultatory findings. Chest X-ray findings during diagnosis of HSV infection were reviewed by a radiologist (RCH) blinded to clinical information on individual patients. Medical diagnoses were obtained from chart descriptors and review. Particular attention was paid to the reasons for ICU stay during the week prior to HSV positivity in respiratory secretions as many patients had multiple comorbidities and prolonged ICU stays. The diagnoses of acute respiratory distress syndrome (ARDS), severe sepsis, and ventilator-associated pneumonia (VAP), were made based on accepted practice standards [Appendix I].

In a number of patients, bronchoscopy was performed and the description of airway abnormalities and available cytological specimens reviewed. BAL was performed with instillation and aspiration of 2–3 aliquots of 60 cc of normal saline in a wedged lung segment of interest. In patients with suspected nosocomial pneumonia, BAL was sent for Gram stain, quantitative bacterial cultures, and HSV PCR.

Approval from Intermountain Health Care institutional review board was obtained for conducting this study. The need for informed consent from patients was waived by the review board due to lack of any patient identifiers.

## Results

All patients with HSV-1 in respiratory secretions diagnosed by qualitative PCR over a five-year period at a tertiary-level multispecialty ICU were analyzed. Between 2003 and 2007, 56% (46/88) of sputum specimens, 31.3% (30/96) of BAL specimens, and 3.3% (7/23) of bronchial washings at the Utah Valley Regional Medical Center yielded HSV by PCR. Of these, 48 HSV-1 positive patients from surgical, medical, and coronary ICUs were identified. All, but two patients were Caucasians. A majority of patients testing positive for HSV were elderly, critically ill, and septic [Table 1]. Unlike previous reports of an increased association in smokers,[6] only 8/48 patients had a smoking history or documented COPD. After excluding patients undergoing cardiac surgery, 11/37 patients had underlying cardiac illnesses, mostly congestive heart failure. Although 85% of the patients were on insulin infusions for tight blood glucose control during their ICU stay, only 17 patients had preexisting diabetes mellitus.

**Table 1:** Demographic data and underlying disease diagnoses

Age (years)	61.9 yrs (24-83yrs)
Sex (Male:Female)	25: 23
APACHE II score on day of HSV diagnosis	31 (15–49)
**Medical diagnoses**	
Severe Sepsis	20/48
Pneumonia (CAP, Aspiration pneumonia)	12 (7, 5)
Severe urosepsis	2
Abdominal sepsis	5
Endocarditis	1
Patients with ARDS	21/48
Neurological problems	6
Cervical cord ischemia	1
Intraventricular bleed	1
Subdural Hematoma	2
Concomitant strokes	2
COPD exacerbation	4
Immunosuppressed patients	15/48
Transplant-related	2
Steroids for COPD exacerbation	3
Steroids for neurological diagnoses	2
Steroids for fibroproliferative ARDS	2
Miscellaneous diagnoses*	6
**Surgical diagnoses**	
Thoracic surgery	
CABG, valve replacement	11
Decortication for empyema	2
Neurosurgery	2
Abdominal surgery (usually for sepsis)	5
Trauma (Traumatic brain injury)	3[2]
**Incidence of chronic diseases**	
Moderate to severe COPD	8/48 (17%)
CHF (EF < 50%) (excluding CABG pts)	11/37 (30%)
Known diabetes	17/48 (35%)

* Ulcerative colitis, IPF exacerbations, and cancer chemotherapy. CAP, community-acquired pneumonia; IPF, idiopathic pulmonary fibrosis; CABG, coronary artery bypass grafting; CHF, congestive heart failure; EF, ejection fraction

The three most common associations for a positive HSV PCR were critical illness (20 patients), steroid use (13 patients), and thoracotomy for coronary artery bypass grafting (CABG) or valve surgery (11 patients) [Table 1]. HSV was identified an average of 13 days after hospitalization, and in intubated patients, after nine days of mechanical ventilation [Table 2]. HSV was detected in respiratory secretions after a mean duration of 11 days of steroid initiation and eight days after thoracotomy [Table 2]. HSV was most often detected in sputum obtained through endotracheal aspiration (30/48 patients) or from BAL samples (19/48 patients). Testing from multiple respiratory sources was done in seven patients [Table 2].

**Table 2:** Timing and method of HSV-1 detection

**Time to HSV-1 detection (days)**	
Following admission	13 days (0–44 days)
Administration of steroids	11.1 days (0–25 days)
Thoracic surgery	8 days (3–15 days)
Following intubation	9.4 days (0–41 days)
Following intubation in septic/	9.4 days (0–32 days)
ARDS patients	
**Method of HSV detection**	
Endotracheal aspirate	30/48 (62.5%)
BAL	19/48 (39.6%)
Bronchial washings	7/48 (14.6%)

Table 3 details clinical findings associated with the presence of HSV in respiratory secretions. 20/48 (42%) of the patients developed orofacial herpes. Of the 18 patients who underwent bronchoscopy, 15 (83%) had findings of tracheobronchial ulcers, erythematous mucosa, and mucosal friability. Cytopathological examination of BAL specimens showed characteristic herpetic inclusions in only 38% of the specimens available for review. A number of patients had either hemorrhagic respiratory secretions (17/48) or excessive sputum production (14/48). Wheezing was found in 25% of the patients and 10 patients were noted to have postextubation stridor for which noninvasive ventilation and/or steroids were used. On *posthoc* review of serial chest radiographs, most patients had either unchanged or worsened chest X-rays on the day of HSV detection. In 29% of patients, definite clinical deterioration was noted during the time of HSV detection; but in the majority, HSV-1 positive patients were significantly ill from underlying morbidities that precluded an accurate assessment of definitive clinical changes.

**Table 3:** Abnormalities suggestive of herpetic reactivation

Specific	Number of patients (%)
Orofacial herpes	20/48 (42)
Hemorrhagic respiratory secretions	17/48 (35)
Bronchoscopic findings of tracheobronchitis	15/18 (83)
Abnormalities on BAL cytology	5/13 (38)
**Nonspecific**	
Wheezing	12/48 (25)
Postextubation stridor	10/36 (28)
Increased respiratory secretions	14/48 (29)
Abnormalities in chest radiographs	
Normal radiograph	0
Unilateral consolidation/patchy opacities	12/48 (25)
Bilateral consolidative/patchy/opacities	19/48 (42)
Perihilar reticular edema	6/48 (12.5)
Atelectatis	10/48 (21)
Radiologic change	
Worsened	21/48 (25)
Unchanged	24/48 (50)
Improved	3/48 (6)
Evidence of clinical deterioration	
Definite	14/48 (29)
Undetermined	31/48 (65)
None	2/48 (4)
Elevation of CRP values	28/28 (100)
Fever (>38.5 °C)	15/48 (31)
Leucocytosis (>11,000 WBC/cu.mm)	32/48 (67)

BAL – Bronchoalveolar lavage; CRP – C-reactive protein; WBC – white blood cell

Of the 48 HSV positive patients, 25 developed nosocomial pneumonia (ventilator-associated pneumonia 23, hospital acquired pneumonia 2) [Table 4]. Causative organisms included both Gram positive and Gram-negative bacteria [Table 4]. The mortality rate was high in patients with HSV, despite specific treatment of herpes simplex and concomitant infections [Table 4]. All patients were treated with intravenous acyclovir at a dose of 5 mg/kg given 2–3 times a day based on their renal function. Although a number of patients had evidence of clinical or radiologic deterioration that improved with antiviral therapy, withdrawal of life support in a number of cases made it difficult to compare mortality rates among HSV-positive groups. The duration of mechanical ventilation in HSV-positive patients was much longer than the average length of stay on the ventilator in our institution (9.3 days for ARDS patients in the year 2007). Post-thoracotomy patients with HSV-1 isolated from respiratory secretions had longer ventilator days as compared to our institutional average (time to first extubation < 10 hours in 2007). Thirty three percent of HSV-1 positive patients required tracheostomy and 61% required placement in a skilled nursing facility following hospital discharge [Table 4].

**Table 4:** Outcomes and hospital course

No of patients on mechanical ventilation	42/48
Duration of mechanical ventilation	
Overall	21.1 days
	(0–85 days)
Patients with sepsis/ARDS	27.7 days
	(5–56 days)
Nonseptic patients	23.3 days
	(1–85 days)
Post-thoracic surgery	15.8 days
	(1–28 days)
No. of patients only on noninvasive ventilation	6/48
No. of patients treated for presumed HAP or	25/48 (52%)
HAP during or after detection of HSV	
Methicillin-sensitive *staphylococcus aureus*	4
Methicillin-resistant *staphylococcus aureus*	3
*Pseudomonas aerogunosa*	6
*Klebsiella pneumoniae*	5
*Enterobacter cloacae*	4
*Stenotrophomonas maltophilia*	1
Beta-hemolytic *streptococci*	1
*Citrobacter freundi*	1
Overall mortality	20/48 (42%)
Mortality based on disease category	
Sepsis/ARDS	7/20 (35%)
Nonseptic patients	13/20 (65%)
No. of patients requiring tracheostomy	16/48 (33%)
Discharge disposition in survivors	
Home	11/28 (39%)
Skilled nursing facility	17/28 (61%)

HAP, hospital-acquired pneumonia; VAP, ventilator-associated pneumonia

## Discussion

In humans, HSV-1 infection is characterized by latent infection in the sensory ganglia of cranial nerves with reactivation under conditions of stress.[1] Stressors that lead to reactivation range from local trauma (dental procedures, sunburn, trigeminal nerve root decompression, etc.) to systemic immunosuppression that especially affects the T-cell immunity (organ or marrow transplantation, HIV infection).[1] Following oropharyngeal reactivation, seeding of the virus into the respiratory and gastrointestinal tract can occur and the potential for herpetic tracheobronchitis and esophagitis exists. However, secondary infections in the respiratory and gastrointestinal tract are reported very infrequently in immunocompetent patients, and visceral disease is not commonly recognized in immunocompetent patients with orofacial herpes.

In the last few decades, a number of reports have highlighted the occurrence of herpetic visceral infections, especially herpetic bronchopneumonitis in relatively nonimmunosuppressed populations. Pulmonary HSV infections were initially recognized in patients with burns[7,8] and allogeneic transplants.[2] A seminal study by Tuxen *et al*, showed that patients with ARDS who tested positive for HSV showed both an increase in duration of ventilatory support and late mortality.[9] Further studies have shown HSV reactivation in patients undergoing thoracotomy[10,11] and in patients with critical illness.[12,13] Critically ill populations that reactivate HSV not only include medically ill septic patients but also surgical and trauma patients.[14,15] One of these studies in critically ill patients established that HSV infection encountered in the ICU occurs due to reactivation of latent virus residing in sensory cranial nerve ganglia rather than from new infection acquired nosocomially.[13] This finding of HSV reactivation in critically ill patients has been explored to understand its impact on outcomes in bigger studies, however, a mortality effect has not been clearly demonstrable.[16,17]

The use of highly sensitive PCR technique has also revolutionized the diagnosis of viral infections, especially HSV1 infections. PCR increases the detection rate of HSV-1 by 44% when compared to the cumbersome culture technique.[18] In addition, PCR is inexpensive and results can be obtained quickly.[3,18] The identification of HSV1 by PCR in the lung, however, may denote lower respiratory tract infection or contamination from oropharyngeal or orofacial herpetic reactivation. Quantitative BAL PCR has been recommended to distinguish herpetic bronchopneumonitis from upper airway contamination;[16,19] however, viral loads that accurately discriminate between contamination and infection are not defined.

The full spectrum of HSV infection of the lower respiratory tract remains to be established. Although initial studies emphasized tracheobronchitis as the main manifestation of herpetic infection,[5,8] a spectrum of abnormalities have been described, ranging from asymptomatic shedding from viral activation in the ninth and tenth cranial nerves,[20] pneumonia,[2,8,11,15] tracheal stenosis,[21] and an effect on lung capillary permeability.[22] This study aimed to identify ICU populations susceptible to herpetic reactivation, to define patterns of HSV respiratory disease, and to understand the impact of HSV disease on hospital course and coinfections.

### Susceptible populations for HSV infection

Although most patients were critically ill during their hospital stay, specific triggers for herpetic reactivation included cardiac surgery (11 patients), steroid use (13 patients), and cranial surgery (2 patients). In the majority, sepsis with and without ARDS contributed to HSV reactivation. Sepsis is well known to be followed by extensive lymphocyte apoptosis[23] that can lead to immunosuppression and possible herpetic reactivation. It is however unclear whether concomitant lung injury is an independent predictor of HSV reactivation, as the majority of patients in our series and prior reports had ARDS.[9,12,13,16] The timing of reactivation in prior studies (12–15 days;[9] 14 days[24]) overlaps with the mean time to detection in our study (13 days).

Apart from surgical trauma due to thoracotomy, steroid therapy appears to be an important trigger for herpetic reactivation.[13,16] Although many patients received empiric stress–dose steroids for management of septic shock, most with HSV reactivation had received higher steroid doses (>0.5 mg/kg/day of prednisone) given for reasons other than adrenal dysfunction. Given the number of hospitalized patients who receive significant doses of steroids for a variety of problems, reports of serious herpetic disease are rare.[25,26]

### Patterns of HSV infection

Most studies analyzing HSV-related respiratory disease have tested for HSV in a setting of suspected VAP.[12,16,27] In this study as well, the impetus to test for HSV was a clinically perceived respiratory deterioration and the concern of nosocomial pneumonia. The majority of patients in our study had abnormal respiratory secretions (hemorrhagic secretions or increased quantity of secretions), abnormal chest radiographs, and increased white blood cell and C-reactive protein values, but fever and changes of severe sepsis were not consistently evident. During diagnosis of HSV infection in the ICU, it was difficult to assign clinical deterioration as being due to herpetic infection because of concomitant comorbidities, especially nosocomial pneumonia. Based on these data, it is difficult to ascribe a unique clinical presentation to herpetic reactivation in ICU patients although the finding of orolabial lesions, hemorrhagic secretions, and wheezing may suggest reactivation of herpetic bronchopneumonitis. Interestingly, 10 HSV positive patients developed postextubation stridor requiring treatment with noninvasive ventilation. Stridor has been described as a complication of HSV-associated tracheitis.[21] Commonly, postextubation stridor in the ICU is treated with steroids that paradoxically may worsen HSV growth.[28]

### Role of cytological diagnosis

In a prospective study done by Luyt *et al*, investigators categorized patients with HSV reactivation into those with and without herpetic bronchopneumonitis, based on findings of herpetic inclusions in BAL or endobronchial samples.[16] Although it is important to separate HSV reactivation in the oropharyngeal region from that in the lower respiratory tract, consistent demonstration of HSV inclusions in respiratory samples is not easily demonstrable. Our study found bronchoscopic abnormalities in the majority of HSV positive patients while only 5/13 (38%) had cytologic evidence of herpetic inclusions on BAL fluid analyses. There were a number of reasons for failure to demonstrate cytologic inclusions in all the BAL specimens. Firstly, cytological specimens included only BAL, not biopsied or brushed mucosa. This has been reported to affect the yield of cytologic inclusions in pathologic specimens.[16] Secondly, immunostaining was not used to demonstrate the finding of HSV in cytologic specimens. In an autopsy study of patients with burns, typical herpetic inclusions were found in none of the 54 hematoxylin and eosin stained lung specimens, however HSV was detected in 50% of these cases by immunostaining.[7] Thirdly, the timing of the bronchoscopy did not coincide with demonstration of HSV in endotracheal aspirate in some of the patients that were intubated for prolonged periods of time. An additional finding that has not been emphasized before has been the scarcity of typical inclusion-laden cells in BAL samples that indicate herpetic bronchopneumonitis. In our review of the available BAL specimens, there were only few cells in each slide that were typical of herpetic inclusions and the finding of herpetic inclusions had been missed in four of the five patients in the initial pathologic evaluation. Nash reported a similar experience in his autopsy series, where the diagnosis of herpetic bronchopnuemonitis was initially overlooked in all but one patient.[8]

### Impact of HSV infection

The majority of patients in our series had prolonged intubation and need for mechanical ventilatory support. Whether this was a consequence of their underlying disease or due to the added morbidity from herpetic reactivation is unknown. Since a number of patients in our study had concomitant nosocomial pneumonia, this could have additionally contributed to prolongation of ICU stay. Besides this study, other studies have also noticed prolonged ventilator stays and an association with bacterial infections.[16,29]

### Study limitations

A major limitation of our study remains the use of the highly sensitive PCR method for diagnosing pulmonary HSV infection. This qualitative PCR technique is unable to distinguish lower airway contamination from oropharyngeal reactivation. However, the decision to test for HSV was based on clinical suspicion of herpetic tracheobronchitis (abnormal respiratory secretions, wheezing, and abnormal bronchoscopy findings) or bronchopneumonia (abnormal respiratory secretions, chest X-ray opacities, and systemic features of infection namely fever, leucocytosis, and increased C-reactive protein values). Even though these clinical criteria were not predefined, there was general consensus among participating intensitivists regarding applicability of these clinical abnormalities to drive testing for HSV. This feature has not been emphasized in prior studies on HSV where testing has been quite arbitrary. Further studies will need to emphasize the clinical abnormalities that trigger HSV testing in order to measure the validity of the PCR and other techniques in denoting clinically significant HSV reactivation. In addition, the lack of predefined clinical criteria to initiate testing for HSV in ICU patients may have resulted in underestimation of the HSV incidence in ICU patients. Since this was a retrospective study, appropriate matched controls were not available for assessing the true impact of HSV reactivation in ICU patients.

## Conclusion

Identification of HSV in respiratory secretions has been vastly improved by the increased availability of PCR that can detect HSV1 in all types of respiratory specimens. The convenience and rapidity of the PCR technique allows early detection in susceptible populations – critically ill, septic patients with lung injury who often require prolonged mechanical ventilation. Steroid use and post-thoracotomy patients may be particularly at risk. Even though detection of HSV has been improved, the separation of mere shedding from disease is clinically challenging. Future studies are required to determine the extent of testing that is required to differentiate bronchial shedding or contamination from upper respiratory tract reactivation versus true herpetic bronchopneumonitis.

## Appendix I

Diagnostic criteria used:

*Acute Respiratory Distress Syndrome*[30]

-  Bilateral radiographic infiltrates

-  A ratio of the partial pressure of oxygen to the fraction of inspired oxygen ≤200

-  No clinical evidence of left atrial hypertension

*Severe Sepsis*[31]

Sepsis and at least one of the following signs of organ hypoperfusion or organ dysfunction: areas of mottled skin; capillary refilling of ≥3 s; urinary output of <0.5 mL/kg for at least 1 hour or renal replacement therapy; lactate > 2 mmol/L; abrupt change in mental status or abnormal EEG findings; platelet count of <100,000 cells/mL or disseminated intravascular coagulation; acute lung injury/ARDS; and cardiac dysfunction (echocardiography).

*Ventilator-associated pneumonia*[32]

Presence of a new or progressing radiographic infiltrate in an intubated patient and acute onset of two of the following: fever (>38 C); leukocytosis (>12000 cells/mm^3^) or neutropenia (<3500 cells/mm^3^); purulent endotracheal secretions. PLUS – ndings of potential pathogenic bacteria from endotracheal aspirate or bronchoalveolar lavage in significant concentrations (>10^4^ bacteria/mm^3^).
